# The effect of endoskeleton on antibiotic impregnated cement spacer for treating deep hip infection

**DOI:** 10.1186/1471-2474-12-10

**Published:** 2011-01-13

**Authors:** Kuo-Ti Peng, Liang-Tseng Kuo, Wei-Hsiu Hsu, Tsan-Wen Huang, Yao-Hung Tsai

**Affiliations:** 1 Division of Sports Medicine, Department of Orthopedic Surgery, Chang Gung Memorial Hospital at Chia Yi, (6 West Section Chia Pu Road), Chia Yi Hsien,(613), Taiwan; 2 Graduate Institute of Clinical Medical Science, College of Medicine, Chang Gung University, (259 Wen-Hwa 1st Road, Kwei-Shan), Tao-Yuan, (333), Taiwan

## Abstract

**Backgrounds:**

A two-stage revision arthroplasty was suggested optimal treatment for deep infections in hip joint. The effect of endoskeleton of cement spacers on the interim function and infection control remains unclear.

**Methods:**

From Jan. 2004 to Dec. 2007, we collected a prospective cohort of consecutive 34 patients who treated with two-stage revision total hip arthroplasty for deep infection of hip joint. In group 1, fifteen patients were treated by a novel design augmented with hip compression screw while nineteen patients were treated by traditional design in group 2.

**Results:**

No fracture of cement spacer occurred in group 1 while 6 cases developed spacer failure in group 2. (p < 0.05) There were significant differences in bodily pain and general health perception between groups (p < 0.05).

**Conclusions:**

Patients being treated for deep infection of hip joint using cement spacer augmented with stronger endoskeleton have lower pain levels and better joint function between stages.

## Background

**Figure 1:**
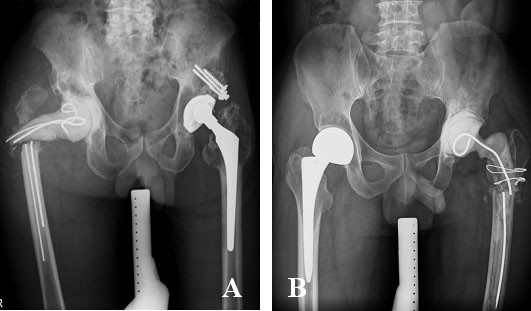
**Fracture of cement spacers may occur in either multiple small diameter Kirschner wires (Figure 1A) or single large diameter Kirschner wire (Figure 1B) **.

A two-stage revision arthroplasty was suggested as the gold standard treatment among many therapeutic alternatives for deep infections in the hip joint[1-6] Various design of antibiotics impregnated cement prosthesis, either custom made or commercially available[7], reported advantages such as effective local antibiotics delivery, continuation of patient mobility, maintenance of limb alignment, and facilitation of re-implantation, contributed to the good functional recovery after revision total hip arthroplasty(THA) [8-12]. However, fracture of the cement spacer occurred which might decrease ambulation ability and leg length discrepancy [10,11,13], and demand an additional surgery for exchange [9] (Figure 1). The introduction of metallic endoskeleton such as Kirschner wires [11,14], Rush rod[8,10], hip compression screws, intramedullary nail[15],and even custom made rod[16] aimed to increase the strength of the construct. Among those designs of endoskeleton, hip compression screw was of particular interest because it was privileged by providing angular stability that augmented the weakest link of the cement spacer [17]. It was possible that mechanical instability, pain, and cement spacer dislocation might be avoided through increasing the strength of these cement spacers [10,11,13].

It was unclear whether different designs of endoskeleton might affect the results of interim function, infection control, and mechanical complications. The purpose of this study was to investigate the effect of antibiotic impregnated cement prosthesis augmented with hip compression screw as compared to those of Kirschner wires in treating infected THA. We hypothesized that the stronger endoskeleton would reduce the incidence of cement prosthesis fracture and increase the functional results.

## Methods

We collected a cohort of consecutive 34 patients who were treated with two-stage revision THA for deep infection of hip joint in Chang-Gung Memorial Hospital at Chia-Yi from January 2004 to December 2007. This study was approved by internal board review of Chang Gung Memorial Hospital (CGMH 98-0330B). Patients were divided into two groups according to the type of endoskeleton for cement spacer. In the study period, fifteen patients were treated with antibiotic impregnated cement spacer augmented with hip compression screw after resection arthroplasty, which were assigned as group 1, whereas nineteen patients were treated with antibiotic impregnated cement spacer augmented with 3.0 Kirschner wires, which were assigned as group 2. The diagnosis of infection was made on clinical examination, presence of a discharge sinus, frank purulent fluid or pus found during on explorative operation, or positive findings on laboratory and histopathologic tests [18].

The first-stage procedure consisted of excision of the sinuses, removal of all implants, hardware, cement, and adequate debridement as well as sequestrectomy for necrotic and infected bone. Three sets of bacterial culture (both aerobic and anaerobic) were taken from the joint fluid, soft tissue and infected bone during debridement. A custom-made metal molds for fabricating the cement prosthesis was developed as previously described [11]. A regimen of 4 gm of vancomycin and 8 gm of piperacillin antibiotic power were mixed with each package of 40 gm of cement polymer (Zimmer Inc., Warsaw, IN). Processed hip compression screw (in group 1) and 3.0 mm Kirschner wires (in group 2) were fabricated as endoskeletons. Specifically, the hip compression screw consisted of 75 mm lag screw and 5-6 holes of side plate (depend on the patient's leg length) was assembled after the side plate was machined to 13 mm in width. The femoral canal was reamed to 13 mm in diameter and the acetabulum was reamed to 56 mm in diameter. The fixation was achieved by manually cementing the cement prosthesis to the proximal part of the host femur. The acetabular component was shaped by a unipolar cup. The patients were encouraged to walk with toe-touch weight-bearing. The hip joint movement was allowed between 0 to 90 degrees as tolerated. The second-stage procedure was performed after the wound healed, along with erythrocyte sedimentation rate and serum C-reactive protein (CRP) both returned to normal range. Two sets of bacterial culture were obtained during revision arthroplasty.

### Evaluation

The fracture of cement spacer was evaluated with anteroposterior and lateral radiographs of the hip and gross assessment for fracture or fragmentation during the second stage procedure. Subjective outcome before second-stage procedure was evaluated with the Medical Outcomes Study Short-Form 36 (SF-36) Health Survey[19]. The SF-36 is a multi-purpose, short-form health survey which yields an 8-scale profile of functional health and well-being scores as well as psychometrically-based physical and mental health summary measures and a preference-based health utility index.

### Statistic analysis

The proportion of cement spacer fracture was compared between groups using Fisher's exact method. Functional outcome was analyzed by Sign rand sum test. Significance was set at 0.05. Re-infection was defined as a recurrence of inflammation with a positive culture or clear radiological and serological evidence of sepsis.

## Results

In group 1, fifteen patients (9 men and 6 women; age 65.9 years, range 36-87 years; body weight 63.5 kg, range 50-74 kg) were included (Table 1) while nineteen patients (13 men and 6 women; age 64.1 years, range 45-80 years; body weight 65.9 kg, range 45-78 kg) were included in group 2 (Table 2). There were no significant differences between groups in demographic data.

**Table 1:** Demographic data in Group 1

**Patient No**.	Sex	Age	Etiology	Side	Pathology	Fracture of DHS augmented cement prosthesis	Revision THA
1	M	36	THA infection	R	Staphy.aureus	No	Yes
2	F	87	Septic hip	L	No growth	No	Yes
3	F	54	Septic hip	L	No growth	No	Yes
4	M	55	Septic hip	R	Staphy.aureus	No	Yes
5	M	87	Septic hip	R	No growth	No	Yes
6	F	84	Septic hip	L	Staphy.aureus	No	Yes
7	M	58	Septic hip	L	Viridans streptococcus	No	No
8	M	44	Septic hip	L	Staphy.aureus	No	Yes
9	M	67	THA infection	R	No growth	No	Yes
10	F	83	THA infection	R	B-Strepto.Gr.B	No	No
11	M	66	THA infection	L	Staphy.aureus	No	No
12	F	72	THA infection	R	No growth	No	Yes
13	M	56	THA infection	L	Strepto. Gr.D	No	Yes
14	F	68	Septic hip	R	No growth	No	Yes
15	M	72	THA infection	R	Staphy.aureus	No	No

**Table 2:** The demographic data in group 2

**Patient No**.	Sex	Age	Etiology	Side	Bacterial Culture Pathology	Fracture of K-pin augmented cement prosthesis	Revision THA
1	M	68	THA infection	R	Peptostreptococcus sp	NO	YES
2	M	57	Septic hip	L	Viridans streptococcus(B)	NO	YES
3	M	80	THA infection	L	No growth	NO	YES
4	M	45	THA infection	R	No growth	YES (spacer neck)	YES
5	M	56	Bipolar infection	R	No growth	NO	YES
6	M	62	THA infection	L	Staphy.aureus	NO	YES
7	F	80	THA infection	R	No growth	YES (spacer neck)	YES
8	M	45	Septic hip	L	Staphy. aeureus	NO	YES
9	F	77	THA infection	R	Staphy.aureus	NO	YES
10	M	71	Septic hip	L	E. Cloacae	YES (spacer neck)	YES
11	F	59	THA infection	R	Staphy. aureus	NO	YES
12	F	77	Septic hip	L	No growth	NO	YES
14	F	73	THA infection	L	Staphy. aureus	NO	YES
15	M	66	THA infection	L	Staphy. aureus	YES (spacer neck)	YES
16	M	46	THA infection	R	No growth	NO	YES
17	M	54	THA infection	R	No growth	YES (spacer neck)	YES
18	M	75	THA infection	R	Staphy. aureus	YES (spacer neck)	YES
19	M	62	THA infection	L	Ps.aeruginosa	NO	YES

**Figure 2:**
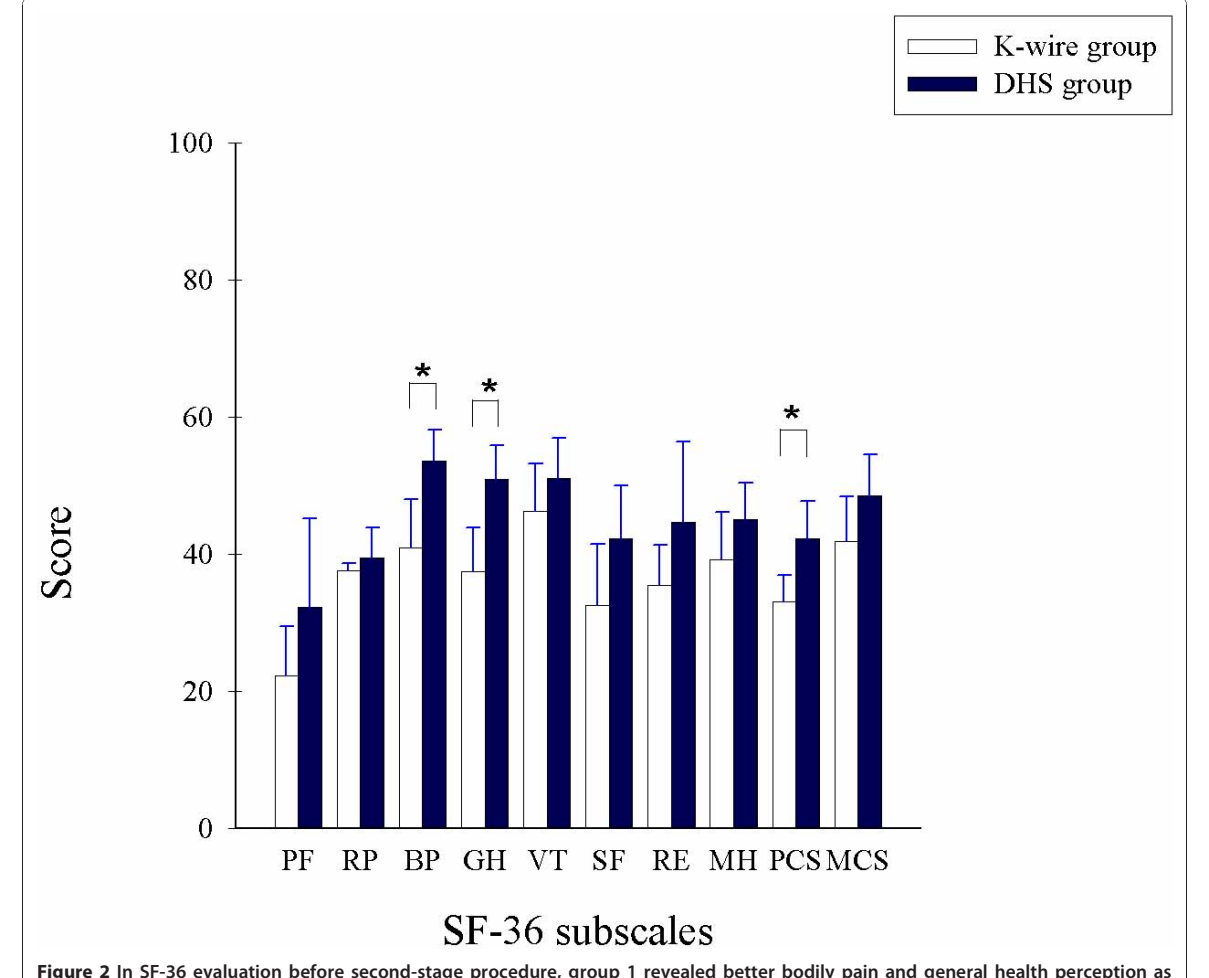
**In SF-36 evaluation before second-stage procedure, group 1 revealed better bodily pain and general health perception as compared to group 2 **. ( bodily pain, group 1 v.s. group 2 = 54 ± 6, v.s. 41 ± 7, p < 0.01; general health perception, group 1 v.s. group 2 = 51 ± 5 v.s. 38 ± 7, p < 0.01) PF: physical functioning, RP: role limitations, BP: bodily pain, GH: general health perceptions, VT: vitality, energy or fatigue, SF: social functioning, RE: role limitations due to emotional problems, MH: general mental health, PCS: physical component status, MCS: mental component status

No fracture of cement spacer occurred in group 1 while 6 cases developed spacer failure in group 2. All fractures occurred over the neck of cement spacers. The differences in proportion of cement spacer fracture between these two groups were statistically significant. (p < 0.05) In SF-36 evaluation, group 1 revealed better bodily pain and general health perception as compared to group 2. (bodily pain, group 1 vs. group 2 = 54 ± 6, vs. 41 ± 7, p < 0.05; general health perception, group 1 vs. group 2 = 51 ± 5 vs. 38 ± 7, p < 0.05) in patient oriented assessment before second stage procedure. (Figure 2)

**Figure 3:**
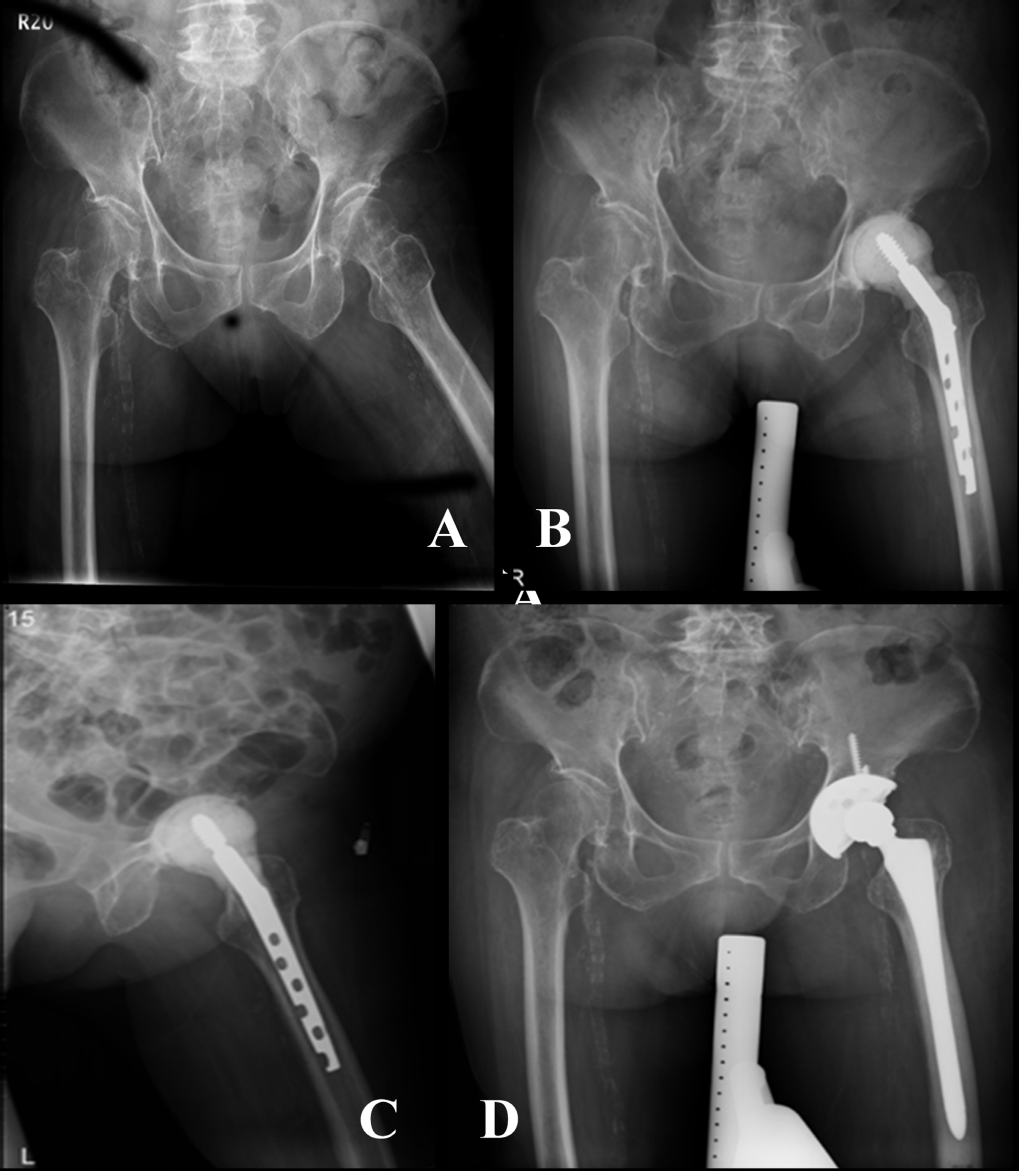
A: A patient with left primary hip septic arthritis; **B-C**: The patient was treated with antibiotic impregnated cement spacer augmented with hip compression screw after resection arthroplasty. **D**: After stabilization of infection, revision total hip arthroplasty is performed.

Interim period between stages averaged 7.2 months (ranged from 5.4 to 9.6 months) for group 1 and 6.7 months (ranged from 4.8 to 11.5 months) for group 2, respectively. Gram-positive microorganism, predominantly Staphylococcus was the most common organism found in the tissue culture during first stage procedure. All cases in our study, both group 1 and group 2, had good infection control after the first-stage procedure and there was no evidence of infection while performed second stage operation. A patient with left primary hip septic arthritis in group 1 was demonstrated. (Figure 3) Three patients refuse revision THA because well tolerance to the cement prosthesis augmented with hip compression screw (case 10, 11 and 15).

## Discussion

The most important finding in the current study was that stronger endoskeleton, such as hip compression screw, in the cement spacer could decrease the cement spacer fracture and hence to improve the interim functional outcome. Fracture of the cement spacer over the neck usually resulted in dislocation of hip joint. The soft tissue envelope of hip joint might then be attenuated. Meanwhile, the superior and posterior acetabular bone stock might also be destroyed. Both consequences increased the difficulties in reconstruction of acetabulum during revision hip arthroplasty. In the current study, no cement facture or dislocation was reported in the group 1 whereas 6 cases developed cement spacer neck fracture in the group 2. These results correlated with the SF-36 evaluation that patients reported better functional results in group 1 as compared to those in group 2.

For treatment of infected THA, a two-stage technique which allows identification of the infecting organism, ascertainment of antibiotic sensitivity and modifying instruction of antibiotic therapy before reimplantation. Antibiotic-impregnated cement was suggested as a safe method in delivering antibiotics with a high initial release and progressively decreases over a period of time to the infection site. Two-stage exchange offers the greater success rate than one-stage exchange with antibiotic-loaded cement in literature [20]. Penner et al. suggested combined antibiotics in the cement can increase the amount and duration of antibiotic elution [21]. On the other hand, literature also suggested that the release of commercially-impregnated antibiotics from hip spacer is significantly increase in the presence of an endoskeleton, whereas the elution of additional incorporated antibiotic is decreased[16]. In the present study, a regimen of 4 gm of vancomycin and 8 gm of piperacillin was added to each package of 40 gm of cement polymer with metallic endoskeleton augmentation. The cultures of tissue obtained at the second stage were negative in all the patients and there were no infections which required reoperation after the second stage. Even though it was demonstrated successful infection control in this study, further investigation in the in vivo elution characteristics of metallic endoskeleton augmented cement spacer was suggested.

The mechanical properties of hip spacers containing a metallic endoskeleton under experimental conditions were investigated in several studies. Kummer et al. reported that hip spacer containing Charnley prosthesis was stronger than that containing Steinmann pins or a short intramedullary nail and was equivalent in strength to the commercial spacer[15]. Another study showed gentamycin-loaded spacers containing double K-wires as endoskeleton showed an average failure load of 1.6 kN[22]. All failures occurred in the upper third of stem. Whereas, Thielen et al. showed that presence of endoskeleton significantly strengthen hip spacers, which tolerated hip joint force even up to 6.0 kN, enough for daily activities under protection[23,24]. Our study, though lacking the result of experimental mechanical test, showed a favorable clinical result with stronger endoskeleton. No case with spacer fracture was demonstrated in group 1.

Moreover, the mechanical strength of cement is not only influenced by the type of antibiotic and atmospheric pressure, but also by the ratio in which the antibiotics are mixed into the cement[25]. There is no consensus at quantitative information regarding the ideal antibiotic/cement ratio, but most surgeons do not exceed a ratio of 10%[26]. On the other hand, previous study showed that there was a trend of decrease in elution of linezoid in the presence of endoskeleton[16]. In this study, a higher local concentration was targeted by regimen of 4 gm of vancomycin and 8 gm of piperacillin in each package of 40 gm of cement polymer, which may lead to reduced mechanical properties. In this concern, we utilized hip compression screw as endoskeleton to enhance strength of hip spacers. Using this novel design with high dose antibiotics, the current study achieve satisfactory infection control and avoid mechanical complications.

Most studies use custom-made, antibiotic-load standardized cement spacer which works like unipolar hemiarthroplasty prosthesis [8-10,12,14]. Using those cement spacers, there is concern of violation of the acetabular bone which might cause pain and further erosion during weight-bearing activity. The current studies provide a simple and effective method to construct a hand-mold antibiotic-load cement spacer with a cement-on-cement articulation. Partial weight-bearing can be allowed and interim function was improved between stages.

Three patients in our study refuse the second-stage surgery because satisfaction of antibiotic impregnated cement prosthesis augmented with hip compression screw. Although full mobility was not possible, most patients reported an acceptable level of pain and adequate function with use of crutches and toe-tough weight-bearing on the involved extremity. These patients have been deemed clinically clear of infection. For patients suffered high-risk multiple medical disease and was considered surgical candidates, the cement spacer augmented with stronger endoskeleton might provide an alternative solution.

Limitations of present study should be addressed. First, number of patients in each group was relatively small. Second, the study design is prospective without randomization. Two groups of patients received therapy in different time period may introduce bias to the results despite of the same orthopedic surgeron. A larger scale of patients with long term follow-up and randomization was warranted.

## Conclusions

In conclusion, this study shows the continued advantages of using antibiotic impregnated cement prosthesis augmented with hip compression screw in two-stage exchange including cost-effective, successful infection eradication, facilitation of reimplantation. Stronger endoskeleton provided strength to reduce fracture of cement prosthesis. Patients being treated for deep joint infections using cement spacer augmented with stronger endoskeleton have lower pain levels and better joint function.

## Competing interests

The authors declare that they have no competing interests.

## Authors' contributions

KTP participated in the design of the study, collected data, performed the statistical analysis and drafted the manuscript. LTK participated in the design of the study, and revised the manuscript. WHH conceived the study, carried out surgeries, and coordinated the research groups. TWH participated in the design of the study and assisted in the surgery. YHT participated in the design of the study and assisted in the surgery. All authors read and approved the final manuscript.

## Pre-publication history

The pre-publication history for this paper can be accessed here:

http://www.biomedcentral.com/1471-2474/12/10/prepub
